# Diagnostic Entropy as an Administrative Measure of Cross‐System Diagnostic Dispersion in Noncommunicable Disease Inpatients: A Methodological Validation Study

**DOI:** 10.1155/jonm/6726900

**Published:** 2026-09-20

**Authors:** Yanhua Liu, Mengxue Zeng, Xingwen Luo, Qin Lin, Xin An, Oratanasataporn Soonthree

**Affiliations:** ^1^ Division of Health, University of Waikato, Hamilton 3216, New Zealand, waikato.ac.nz; ^2^ West China School of Nursing, Sichuan University, Chengdu 610093, China, scu.edu.cn; ^3^ Department of Thoracic Surgery, West China Hospital, Sichuan University, Chengdu 610093, China, scu.edu.cn; ^4^ Department of Emergency Medicine, The Third People’s Hospital of Yibin, Yibin 644002, China; ^5^ Shulan International Medical College, Zhejiang Shuren University, Hangzhou Zhejiang, 310015, China, zjsru.edu.cn

**Keywords:** administrative data, care coordination, diagnostic entropy, hospital length of stay, multimorbidity, nursing workload

## Abstract

**Background:**

Existing comorbidity indices such as the Charlson Comorbidity Index (CCI) summarise comorbidity burden but do not capture how a patient’s diagnoses are distributed across clinical speciality domains, a dimension hypothesised to relate to cross‐system care coordination demands. This gap limits their utility for nursing workforce planning in settings with high multimorbidity prevalence.

**Aim:**

This study aimed to provide preliminary evidence for the construct validity of diagnostic entropy as an administrative measure of cross‐system diagnostic dispersion, by testing its incremental predictive value for prolonged hospitalisation, beyond diagnosis count and the CCI.

**Methods:**

A retrospective methodological validation study used inpatient discharge records from a tertiary hospital in southwest China, 2023–2025 (*n* = 31,470). Diagnostic entropy was calculated using the Shannon entropy formula applied to ICD‐10 chapter distributions across each patient’s valid diagnoses. Four nested binary logistic regression models were fitted with LOS > 10 days as the primary outcome. Four sensitivity analyses examined robustness to extreme LOS values, coding inclusion rules, insurance‐type stratification and repeat admissions from the same patient.

**Results:**

Diagnostic entropy had a mean of 1.746 (SD: 0.569); 72.3% of patients were in the high‐entropy group (*H* ≥ 1.5). Entropy showed low‐to‐moderate correlation with the CCI (Spearman *r* = 0.304, *p* < 0.001) and moderate correlation with diagnosis count (*r* = 0.544, *p* < 0.001), suggesting that entropy captures a related but nonequivalent aspect of comorbidity‐related complexity. After controlling for demographic characteristics, diagnosis count and CCI, entropy remained independently associated with prolonged hospitalisation (OR = 1.112, 95% CI: 1.072–1.153, *p* < 0.001), with an incremental Nagelkerke *R* ^2^ = 0.0014. This association was evident primarily among patients with seven or more diagnoses and was not statistically significant among patients with fewer diagnoses. High‐entropy patients had longer stays, higher costs and higher rates of prolonged hospitalisation compared to low‐entropy patients. Sensitivity analyses produced consistent results (OR range: 1.103–1.126).

**Conclusions:**

These findings contribute discriminant evidence toward the construct validity of diagnostic entropy as an administrative measure of cross‐system diagnostic dispersion. Whether this dispersion translates into greater nursing coordination burden remains a hypothesis requiring prospective validation.

**Implications for Nursing Management:**

Diagnostic entropy, calculated from routinely collected discharge diagnoses, may offer nurse managers a low‐cost, scalable indicator of cross‐system diagnostic dispersion among NCD inpatients. Wards with a higher proportion of high‐entropy patients could potentially require greater coordination across speciality teams, though this application awaits prospective, multicentre validation linking entropy to nursing‐sensitive outcomes.

## 1. Introduction

Multimorbidity, the co‐occurrence of two or more chronic conditions, has become one of the defining challenges of contemporary health systems. Global pooled prevalence estimates vary by region, ranging from approximately 35% in Asia to over 45% in South America and over 43% in North America [1, 2], and continue to rise universally. Noncommunicable diseases (NCDs) now account for the majority of inpatient admissions in Chinese secondary and tertiary hospitals [3]. Multimorbidity is consistently associated with higher admission rates, prolonged length of stay (LOS) and greater inpatient mortality [4, 5]. Nurses occupy a central role in managing these patients, not only through clinical monitoring but also through the ongoing coordination of care across multiple medical specialities [6]. As NCD prevalence increases, quantifying this coordination burden has become a practical priority for nursing workforce planning.

The Charlson Comorbidity Index (CCI) and the Elixhauser comorbidity measure are the most widely used tools for summarising a patient’s comorbidity profile from administrative data [7, 8]. Both indices were developed primarily to predict in‐hospital mortality; their core logic revolves around identifying present high‐risk conditions and weighting them according to their relative contribution to mortality risk [9]. This design makes them effective measures of overall comorbidity burden, but the weighting scheme performs less reliably for nonmortality outcomes [10]. A more fundamental limitation, in the context of nursing management, is that these indices treat comorbidity as a list of conditions to be summed and weighted, without regard to how those conditions are distributed across clinical systems.

Consider two patients with similar Charlson scores. The first has multiple diagnoses all within the circulatory system, potentially involving a narrower range of clinical expertise. The second has diagnoses spanning circulatory, respiratory, endocrine, renal and mental health domains, potentially involving a broader range of clinical expertise and coordination activities, including management of differing treatment protocols and monitoring of diagnostic workups. Existing comorbidity indices assign these two patients comparable scores, yet their nursing coordination demands may plausibly differ. High clinical dispersion increases the potential for care fragmentation—the uncoordinated delivery of care across multiple providers—which is well‐documented to prolong hospitalisation, elevate costs and increase readmission risk [11–13]. However, an administrative metric to capture this cross‐system diagnostic dispersion remains lacking.

Shannon entropy, originally developed in information theory, offers a natural mathematical framework for quantifying such distributional dispersion. It has been applied in clinical medicine to quantify variability in heart rate [14], tumour heterogeneity [15] and microbiome composition. Its application to administrative diagnostic data includes quantifying diagnostic diversity across age and sex strata [16] and evaluating ICD coding transitions [17], establishing that entropy can be meaningfully computed from ICD chapter distributions. However, systematic validation of diagnostic entropy as an independent predictor of care outcomes, after controlling for both diagnostic volume and established comorbidity indices, has not been established in NCD inpatient cohorts.

In the present study, we propose diagnostic entropy, calculated from the distribution of ICD‐10 chapter codes across a patient’s diagnoses, as an administrative‐data measure of cross‐system diagnostic dispersion. This measure targets a dimension of complexity that diagnosis count and the CCI do not directly capture: whereas diagnosis count reflects diagnostic volume and the CCI reflects weighted severity, diagnostic entropy is proposed to capture how diagnoses are dispersed across clinical speciality domains. We hypothesise that this dispersion may be relevant to cross‐system care coordination demands, an interpretation that requires direct validation against nursing workload indicators in future research. This study aimed to provide preliminary evidence for the construct validity of diagnostic entropy by examining whether it is independently associated with prolonged hospitalisation, beyond both diagnosis count and the CCI in a cohort of 31,470 NCD inpatients.

## 2. Methods

### 2.1. Study Design and Setting

This was a retrospective methodological validation study conducted at Yibin Third People’s Hospital, a nonprofit tertiary Grade A general hospital in Yibin, Sichuan Province, southwest China, established in 1951. The hospital had 750 approved and 810 operational beds during the study period, with approximately 38,700 discharges annually. Cardiovascular medicine, the speciality most relevant to the largest primary diagnosis category in this study, is designated a municipal‐level key clinical speciality. The hospital serves a mixed urban and rural catchment area centred on the municipality, consistent with the substantial proportion of rural residents (61.6%) observed in the study sample. Inpatient medical record data were extracted from the hospital information system for three consecutive years (2023–2025).

### 2.2. Participants

All adult inpatients with a primary diagnosis falling within four major NCD categories were included: circulatory diseases (ICD‐10 Chapter I), respiratory diseases (Chapter J), neoplasms (Chapter C) and endocrine diseases (Chapter E). Inclusion was based solely on the ICD‐10 code of the primary diagnosis at discharge, regardless of the admitting department or ward. The hospital recorded approximately 116,000 total discharges over the 3‐year study period (2023–2025). No records were excluded for missing primary diagnosis. No additional exclusions were applied beyond the prespecified eligibility criteria described above. After restricting to the four NCD categories defined above, the final sample comprised 31,470 records. Because 29.2% of records represented repeat admissions from the same patient (22,282 unique patients across 31,470 admissions), the analytical sample comprised admission‐level records; the resulting nonindependence was addressed in the statistical analysis as follows.

### 2.3. Diagnostic Entropy Calculation

Diagnostic entropy was calculated using the Shannon entropy formula: *H* = ∑*p*_*i*_ × log_2_(*p*_*i*_), where *p*_*i*_ represents the proportion of diagnoses belonging to ICD‐10 Chapter I among all valid diagnoses for a given patient [16]. Base‐2 logarithms were used, consistent with standard information‐theoretic convention, such that entropy is expressed in bits; the choice of logarithm base does not affect the relative ordering of entropy values across patients, only the scale. No duplicate ICD‐10 codes were observed within individual patient records across the diagnosis fields used in this study, so no additional deduplication step was required prior to entropy calculation. A complete list of variable definitions, roles and coding schemes for all models is provided in Supporting Table S1.

Raw (non‐normalized) Shannon entropy was used rather than normalized alternatives (e.g., entropy divided by the maximum possible value, or the effective number of systems, 2^*H*^), as chapter co‐occurrence is not uniform across NCD inpatients and normalization would implicitly assume otherwise. Full justification is provided in Supporting Note S1.

Each patient record contained up to 16 diagnosis fields (one primary and 15 secondary diagnoses). Primary and secondary diagnoses were weighted equally in the entropy calculation; no additional weight was assigned to the primary diagnosis. This decision reflects the study’s focus on the overall distribution of a patient’s diagnostic profile across clinical systems, rather than the relative clinical priority of individual diagnoses, which is not consistently recorded in administrative coding practice. ICD‐10 Chapter Z (factors influencing health status) and Chapter R (symptoms and signs) were excluded from entropy calculation, as they do not represent definitive disease diagnoses; the robustness of this exclusion rule was tested in a sensitivity analysis. The chapter‐level granularity was chosen deliberately: ICD‐10 chapters provide a reproducible administrative approximation of broad diagnostic system domains that may relate to, but do not directly represent, speciality involvement, making chapter‐level entropy a conceptually appropriate starting point for measuring cross‐speciality diagnostic dispersion, which we hypothesise may relate to care coordination demands. A patient with all diagnoses within one chapter receives *H* = 0, indicating a single‐chapter diagnostic distribution; a patient with diagnoses distributed across multiple chapters receives a higher entropy value, reflecting greater cross‐system dispersion. For descriptive purposes (Table 1), patients were further grouped into four entropy bands, with the *H* ≥ 1.5 threshold approximating diagnoses distributed roughly evenly across three or more chapters (log_2_3 ≈ 1.585).

**TABLE 1:** Baseline characteristics of the study sample, stratified by the diagnostic entropy group (*n* = 31,470).

Characteristic	Overall *N* = 31,470^1^	*H* = 0 *N* = 804^1^	0 < *H* < 1.0 *N* = 2521^1^	1.0 ≤ *H* < 1.5 *N* = 5379^1^	*H* ≥ 1.5 *N* = 22,766^1^	*p* value^2^
Age (years)	69 (59, 76)	59 (50, 69)	64 (55, 72)	68 (58, 74)	70 (60, 77)	< 0.001
Sex						0.057
Female	14,649 (47%)	360 (45%)	1126 (45%)	2465 (46%)	10,698 (47%)	
Male	16,821 (53%)	444 (55%)	1395 (55%)	2914 (54%)	12,068 (53%)	
Insurance type						0.072
UEBMI	5154 (16%)	128 (16%)	458 (18%)	855 (16%)	3713 (16%)	
URRBMI	26,316 (84%)	676 (84%)	2063 (82%)	4524 (84%)	19,053 (84%)	
Admission route						< 0.001
Nonemergency	21,587 (69%)	594 (74%)	1862 (74%)	3854 (72%)	15,277 (67%)	
Emergency	9883 (31%)	210 (26%)	659 (26%)	1525 (28%)	7489 (33%)	
Residence						0.049
Urban	12,071 (38%)	292 (36%)	1018 (40%)	2101 (39%)	8660 (38%)	
Rural	19,399 (62%)	512 (64%)	1503 (60%)	3278 (61%)	14,106 (62%)	
Length of stay (days)	8.0 (6.0, 10.0)	6.0 (3.0, 8.0)	7.0 (4.0, 9.0)	7.0 (5.0, 9.0)	8.0 (6.0, 10.0)	< 0.001
Total cost (CNY)	5227 (3549, 8117)	3979 (2404, 6629)	4265 (2752, 7101)	4903 (3381, 7657)	5453 (3728, 8413)	< 0.001
LOS > 10 days	7022 (22%)	107 (13%)	375 (15%)	950 (18%)	5590 (25%)	< 0.001
In‐hospital mortality						0.002
Survived	31,296 (99%)	801 (100%)	2515 (100%)	5362 (100%)	22,618 (99%)	
Died	174 (0.6%)	3 (0.4%)	6 (0.2%)	17 (0.3%)	148 (0.7%)	
CCI	3.00 (1.00, 4.00)	0.00 (0.00, 2.00)	1.00 (0.00, 3.00)	2.00 (1.00, 3.00)	3.00 (2.00, 4.00)	< 0.001
Diagnosis count	8.0 (6.0, 10.0)	2.0 (1.0, 3.0)	5.0 (3.0, 6.0)	7.0 (5.0, 8.0)	8.0 (6.0, 11.0)	< 0.001
Diagnostic entropy	1.84 (1.45, 2.16)	0.00 (0.00, 0.00)	0.81 (0.72, 0.92)	1.36 (1.15, 1.38)	1.99 (1.77, 2.25)	< 0.001
Year						< 0.001
2023	10,840 (34%)	249 (31%)	736 (29%)	1837 (34%)	8018 (35%)	
2024	10,388 (33%)	274 (34%)	885 (35%)	1755 (33%)	7474 (33%)	
2025	10,242 (33%)	281 (35%)	900 (36%)	1787 (33%)	7274 (32%)	

*Note:* Values are presented as median (Q1 and Q3) for continuous variables and *n* (%) for categorical variables. CNY, Chinese Yuan.

Abbreviations: UEBMI, Urban Employee Basic Medical Insurance; URRBMI, Urban and Rural Resident Basic Medical Insurance; LOS, length of stay; CCI, Charlson Comorbidity Index.Entropy groups were defined as *H* = 0 (single‐system diagnoses), 0 < *H* < 1.0, 1.0 ≤ H < 1.5 and *H* ≥ 1.5 (high‐entropy).*p* values were calculated using the Kruskal–Wallis rank‐sum test for continuous variables and Pearson’s chi‐squared test for categorical variables.

### 2.4. Charlson Comorbidity Index

The CCI was calculated using the Quan et al. (2005) ICD‐10 mapping [8], implemented via the comorbidity package (Version 1.1.0) in R [18]. Weighted scores were derived using the Quan weighting scheme [19].

### 2.5. Other Variables

Diagnosis count was defined as the total number of valid ICD‐10 codes per patient (observed range 1–16). Age was standardised to a z‐score. Sex was coded as male = 1. Insurance type was coded as Urban and Rural Resident Basic Medical Insurance (URRBMI) = 1 and Urban Employee Basic Medical Insurance (UEBMI) = 0. Emergency admission, rural residence, primary diagnosis category and year were included as covariates. The primary outcome was prolonged hospitalisation, defined as LOS > 10 days, corresponding to the 75th percentile of the sample distribution (22.3% of patients). Total hospitalisation cost was examined descriptively as a secondary outcome. In‐hospital mortality (*n* = 174, 0.55%) was examined as an exploratory outcome only, given limited events per variable.

### 2.6. Statistical Analysis

Spearman rank correlations were calculated between diagnostic entropy, CCI and diagnosis count to confirm that entropy captures a dimension related to but nonequivalent to both severity and volume of diagnoses [20]. Four nested binary logistic regression models were fitted with LOS > 10 days as the outcome. Model 1 included demographic and clinical covariates only. Model 2 added diagnosis count to isolate the effect of diagnostic volume. Model 3 further added CCI to control for disease severity. Model 4 added diagnostic entropy, representing the core test of incremental predictive value. The final model (Model 4) took the following form: logit(P(LOS > 10 days)) = *β*_0_ + *β*_1_(Age_std) + *β*_2_(Male) + *β*_3_(URRBMI) + *β*_4_ (Emergency) + *β*_5_(Rural) + *β*_6_(Diag_J) + *β*_7_(Diag_C) + *β*_8_ (Diag_E) + *β*_9_(Year2024) + *β*_10_(Year2025) + *β*_11_(Diagnosis count) + *β*_12_(CCI) + *β*_13_(Entropy_std). Full variable definitions, roles and coding are provided in Supporting Table S1. Entropy was standardised before entry into regression models; the resulting OR reflects the change in odds per one standard deviation increase in entropy. Multicollinearity was assessed using variance inflation factors (VIFs), with a threshold of VIF < 5. Model fit was assessed using AUC and Nagelkerke *R* ^2^, and the incremental *R* ^2^ from Model 3 to Model 4 quantified the additional explanatory contribution of entropy.

Four sensitivity analyses were conducted. S1 excluded patients with LOS > 30 days (*n* = 148 excluded) to assess the influence of extreme values. S2 recalculated entropy including Z and R chapter codes to test the robustness of the coding exclusion rule. S3 stratified the main analysis by insurance type (URRBMI vs. UEBMI) to examine consistency across subgroups. S4 restricted the sample to each patient’s first admission to address potential nonindependence from repeat admissions by the same patient, and standard errors were additionally estimated using cluster‐robust estimation at the patient level as a complementary check. All analyses were conducted in R Version 4.5.3. Statistical significance was set at *p* < 0.05.

### 2.7. Ethics

This study was approved by the Ethics Committee of Yibin Third People’s Hospital (approval number: 2025042) and the Human Research Ethics Committee (approval number: HREC(Health)2025#83). Patient data were fully anonymised prior to analysis. Individual informed consent was waived in accordance with the retrospective nature of the study.

## 3. Results

### 3.1. Sample Description

A total of 31,470 NCD inpatients were included across three years (2023: *n* = 10,840; 2024: *n* = 10,388; 2025: *n* = 10,242). The median age was 69 years (IQR: 59 and 76), and 53.5% were male. Most patients were covered by URRBMI (83.6%), and 31.4% were admitted via emergency. Rural residents accounted for 61.6% of the sample. The most common primary diagnosis category was circulatory disease (Chapter I), followed by respiratory disease (Chapter J), neoplasms (Chapter C) and endocrine disease (Chapter E). Baseline characteristics of the sample, stratified by diagnostic entropy group, are presented in Table 1.

### 3.2. Entropy Distribution

**FIGURE 1:**
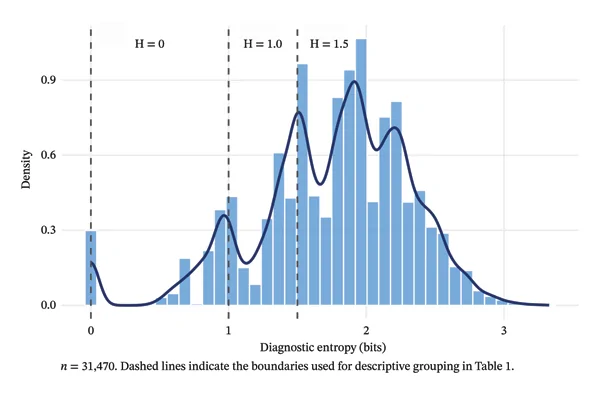
Distribution of diagnostic entropy across the study sample (*n* = 31,470). *Note.* The histogram shows the frequency distribution of entropy values, with a kernel density curve overlaid. Vertical dashed lines indicate the boundaries used for descriptive grouping in Table 1 (*H* = 0, *H* = 1.0 and *H* = 1.5). The distribution is left‐skewed, with the majority of patients concentrated at higher entropy values, reflecting a predominance of cross‐system diagnostic dispersion in this NCD inpatient population.

**FIGURE 2:**
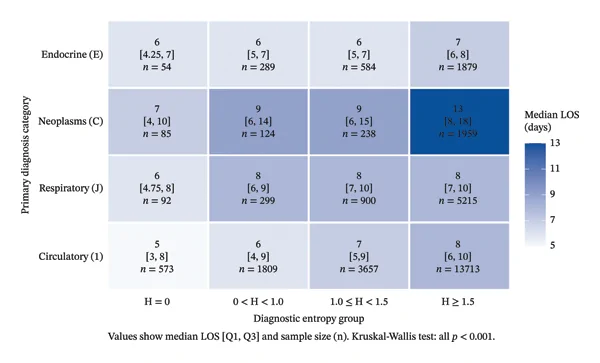
Median LOS by primary diagnosis category and diagnostic entropy group. *Note.* Each cell shows the median LOS with interquartile range in square brackets and the cell sample size (*n*). Colour intensity corresponds to median LOS, with darker shades indicating longer stays. Primary diagnosis categories are ordered along the vertical axis: Circulatory disease (Chapter I), respiratory disease (Chapter J), neoplasms (Chapter C) and endocrine disease (Chapter E). Diagnostic entropy groups are ordered along the horizontal axis: *H* = 0, 0 < *H* < 1.0, 1.0 ≤ *H* < 1.5 and *H* ≥ 1.5. The figure demonstrates a monotonic association between higher entropy and longer LOS across all four primary diagnosis categories, with the highest median stay (13 days) observed among high‐entropy neoplasm patients. Kruskal–Wallis tests across entropy groups within each diagnosis category yielded *p* < 0.001 in all four categories.

As shown in Table 1 and visualised in Figure 1, diagnostic entropy had a mean of 1.746 (median: 1.837, SD: 0.569). Most patients fell in the high‐entropy group: 72.3% had *H* ≥ 1.5 (*n* = 22,766), while only 2.6% had *H* = 0 (*n* = 804), indicating single‐system diagnoses. Compared to the two lowest entropy groups combined (*H* = 0 and 0 < *H* < 1.0, *n* = 3325), patients in the high‐entropy group had longer stays (median 8 vs. 6 days, *p* < 0.001), higher total costs (median CNY 5453 vs. 4,214, *p* < 0.001) and a higher proportion of LOS > 10 days (24.6% vs. 14.5%). In‐hospital mortality was also higher in the high‐entropy group (0.65% vs. 0.27%). Insurance distribution was similar across entropy groups (URRBMI: 83.7% vs. 82.4%), suggesting entropy is not strongly confounded by insurance type. The gradient in LOS across increasing entropy groups within primary diagnosis categories is visualised in Figure 2.

### 3.3. Correlation Analysis

Table 2 presents the Spearman correlations among diagnostic entropy, the CCI and diagnosis count. Diagnostic entropy showed low‐to‐moderate correlation with the CCI (Spearman *r* = 0.304, *p* < 0.001), consistent with related but nonequivalent constructs. Entropy showed a moderate correlation with diagnosis count (*r* = 0.544, *p* < 0.001), justifying the inclusion of diagnosis count as a separate control layer in Model 2.

**TABLE 2:** Spearman rank correlations between diagnostic entropy, the CCI and diagnosis count (*n* = 31,470).

Variable pair	Spearman r	*p* value	Interpretation
Diagnostic entropy vs. CCI	0.304	< 0.001	Low‐to‐moderate correlation; entropy and CCI measure related but nonequivalent dimensions
Diagnostic entropy vs. diagnosis count	0.544	< 0.001	Moderate correlation; diagnosis count controlled separately in Model 2
CCI vs. diagnosis count	0.459	< 0.001	Moderate correlation; reference

*Note:* All correlations were statistically significant at *p* < 0.001. The low‐to‐moderate correlation between diagnostic entropy and the CCI (*r* = 0.304) indicates that the two measures capture related but nonequivalent dimensions of comorbidity‐related complexity. The moderate correlation between entropy and diagnosis count (*r* = 0.544) justified the inclusion of diagnosis count as a separate control variable in Model 2.

Abbreviation: CCI = Charlson Comorbidity Index.

### 3.4. Four Nested Models

Table 3 presents the four nested logistic regression models predicting LOS > 10 days. Model 1 included demographic variables only (AUC = 0.680, Nagelkerke *R* ^2^ = 0.119). Adding diagnosis count in Model 2 improved model fit (AUC = 0.703, *R* ^2^ = 0.147), and diagnosis count was independently associated with prolonged LOS (OR = 1.120, 95% CI: 1.110–1.130, *p* < 0.001). Model 3 further added CCI (AUC = 0.703, *R* ^2^ = 0.148). In Model 4, diagnostic entropy remained independently associated with prolonged LOS after controlling for diagnosis count and CCI (OR = 1.112, 95% CI: 1.072–1.153, *p* < 0.001), with an incremental *R* ^2^ of 0.0014 over Model 3. Model discrimination, as measured by AUC, improved only marginally from Model 3 to Model 4 (0.703 to 0.704). The maximum VIF across all variables in Model 4 was 2.04, indicating no multicollinearity concern.

**TABLE 3:** Four nested binary logistic regression models predicting prolonged hospitalisation (LOS > 10 days) in NCD inpatients (*n* = 31,470).

Variable	Model 1	Model 2	Model 3	Model 4
Age (standardized)	1.114 (1.082–1.147)^∗∗∗^	0.976 (0.946–1.008)	0.982 (0.952–1.014)	0.982 (0.952–1.014)
Sex (male)	1.180 (1.115–1.249)^∗∗∗^	1.156 (1.091–1.224)^∗∗∗^	1.173 (1.107–1.243)^∗∗∗^	1.174 (1.109–1.244)^∗∗∗^
Insurance (URRBMI)	0.835 (0.771–0.905)^∗∗∗^	0.812 (0.749–0.881)^∗∗∗^	0.817 (0.754–0.887)^∗∗∗^	0.822 (0.758–0.892)^∗∗∗^
Emergency admission	1.710 (1.612–1.814)^∗∗∗^	1.650 (1.554–1.752)^∗∗∗^	1.647 (1.551–1.749)^∗∗∗^	1.640 (1.544–1.741)^∗∗∗^
Rural residence	0.976 (0.918–1.038)	0.961 (0.903–1.023)	0.956 (0.899–1.018)	0.956 (0.898–1.018)
Respiratory disease (J)	0.838 (0.780–0.900)^∗∗∗^	1.010 (0.938–1.087)	1.043 (0.967–1.124)	0.997 (0.924–1.076)
Neoplasms (C)	5.967 (5.449–6.536)^∗∗∗^	7.058 (6.427–7.755)^∗∗∗^	8.008 (7.202–8.910)^∗∗∗^	7.522 (6.750–8.387)^∗∗∗^
Endocrine disease (E)	0.276 (0.235–0.323)^∗∗∗^	0.239 (0.203–0.280)^∗∗∗^	0.237 (0.201–0.278)^∗∗∗^	0.239 (0.203–0.280)^∗∗∗^
Year 2024	0.855 (0.800–0.914)^∗∗∗^	0.841 (0.786–0.899)^∗∗∗^	0.840 (0.785–0.898)^∗∗∗^	0.846 (0.790–0.904)^∗∗∗^
Year 2025	0.671 (0.626–0.719)^∗∗∗^	0.671 (0.626–0.719)^∗∗∗^	0.671 (0.625–0.719)^∗∗∗^	0.677 (0.631–0.726)^∗∗∗^
Diagnosis count	—	1.120 (1.110–1.130)^∗∗∗^	1.132 (1.121–1.144)^∗∗∗^	1.112 (1.099–1.125)^∗∗∗^
CCI	—	—	0.961 (0.946–0.976)^∗∗∗^	0.962 (0.947–0.977)^∗∗∗^
**Diagnostic entropy (per SD)**	**—**	**—**	**—**	**1.112 (1.072–1.153)** ^**∗∗∗**^
AUC	0.680	0.703	0.703	0.704
Nagelkerke *R* ^2^	0.119	0.147	0.148	0.150
Max VIF	1.27	1.28	1.49	2.04

*Note:* Values are odds ratios with 95% confidence intervals in parentheses, reported to three decimal places. Model 1 included demographic and clinical covariates only; Models 2–4 sequentially added diagnosis count, CCI and standardised diagnostic entropy. Reference categories: Sex = female; Insurance = UEBMI; Admission = nonemergency; Residence = urban; Primary diagnosis = circulatory disease (Chapter I); Year = 2023. AUC: 0.680/0.703/0.703/0.704; Nagelkerke *R* ^2^: 0.119/0.147/0.148/0.150 (Models 1–4); incremental *R* ^2^ from Model 3 to Model 4 = 0.0014, based on unrounded estimates. Maximum VIF: 1.27/1.28/1.49/2.04 (Models 1–4), well below the conventional threshold of 5. Standard errors in Model 4 were additionally verified using cluster‐robust estimation (clustered by patient); results were materially unchanged (see main text). Bold values indicate the primary result of interest (diagnostic entropy in the fully adjusted model, Model 4).

^∗∗∗^ *p* < 0.001.

^∗∗^ *p* < 0.01.

^∗^ *p* < 0.05.

An unexpected finding in the fully adjusted model was the inverse association between CCI and prolonged hospitalisation; a post hoc case‐mix analysis was therefore conducted to examine this pattern more closely. When the sample was stratified by the presence of a neoplasm diagnosis, the CCI–LOS association remained negative in both subgroups (nononcology patients: OR = 0.972, 95% CI: 0.954–0.990, *p* = 0.003; oncology patients: OR = 0.939, 95% CI: 0.912–0.967, *p* < 0.001). Oncology patients had the highest median CCI (4 vs. 3) and longest median LOS (12 vs. 7 days) in the sample, yet the weakest raw correlation between CCI and LOS (Spearman *r* = 0.042 vs. 0.176 among nononcology patients).

### 3.5. Sensitivity Analyses

Sensitivity analyses yielded estimates broadly consistent in direction and magnitude with the primary analysis. After excluding patients with LOS > 30 days (S1, *n* = 31,322), the entropy OR was 1.103 (95% CI: 1.063–1.144, *p* < 0.001). When Z and R chapter codes were included in the entropy calculation (S2), the OR was 1.122 (95% CI: 1.082–1.163, *p* < 0.001). In the insurance‐stratified analysis (S3), the association was consistent among URRBMI patients (OR = 1.121, 95% CI: 1.078–1.167, *p* < 0.001) but did not reach statistical significance among UEBMI patients (OR = 1.052, 95% CI: 0.960–1.152, *p* = 0.279), likely reflecting limited statistical power in the smaller UEBMI subgroup (*n* = 5154).

After accounting for within‐patient clustering, the association between diagnostic entropy and prolonged LOS remained materially unchanged (cluster‐robust OR = 1.112, 95% CI: 1.070–1.156, *p* < 0.001). Restricting the sample to each patient’s first admission (S4, *n* = 22,282) yielded a similar, slightly larger estimate (OR = 1.126, 95% CI: 1.078–1.176, *p* < 0.001), confirming that the main finding was not driven by within‐patient correlation across repeat admissions.

### 3.6. Stratified Analysis by Diagnosis Count

To examine whether the entropy effect reflects diagnostic dispersion rather than diagnostic volume alone, a fully adjusted model was fitted separately within five diagnosis‐count strata using within‐stratum standardised entropy (Table 4). The association between entropy and prolonged LOS was not statistically significant among patients with 1–3 diagnoses (OR = 1.089, *p* = 0.177) or 4–6 diagnoses (OR = 1.026, *p* = 0.397), but was significant and consistent in magnitude across all three higher strata (7–9, 10–12 and ≥ 13 diagnoses; ORs 1.12–1.14, all *p* < 0.01).

**TABLE 4:** Association between diagnostic entropy and prolonged hospitalisation, stratified by diagnosis count.

Diagnosis count stratum	*n*	OR (entropy)	95% CI	*p*
1–3	2453	1.089	0.963–1.235	0.177
4–6	8808	1.026	0.967–1.089	0.397
7–9	10,800	1.139	1.082–1.200	< 0.001
10–12	6058	1.133	1.064–1.207	< 0.001
≥ 13	3351	1.117	1.032–1.210	0.006

*Note:* Odds ratios (ORs) reflect the association between within‐stratum standardised diagnostic entropy and prolonged hospitalisation (length of stay > 10 days), adjusted for age, sex, insurance type, emergency admission, rural residence, primary diagnosis category, year and the Charlson Comorbidity Index (CCI). Diagnosis count was not included as a covariate within strata because stratification restricts the range of diagnosis count directly. Entropy was standardised separately within each stratum; the OR reflects the change in odds per one within‐stratum standard deviation increase in entropy.

Taken together, the analyses indicate a statistically robust but small association between diagnostic entropy and prolonged hospitalisation that was not fully explained by diagnosis count or CCI.

## 4. Discussion

This study examined whether diagnostic entropy, as a measure of cross‐system diagnostic dispersion, is independently associated with prolonged hospitalisation beyond both diagnosis count and the CCI in NCD inpatients, as a test of its construct validity. After controlling for demographic characteristics, diagnosis count and CCI simultaneously, diagnostic entropy remained independently associated with LOS > 10 days (OR = 1.112, 95% CI: 1.072–1.153, *p* < 0.001).

The magnitude of the entropy effect warrants careful interpretation. The OR of 1.112 per standard deviation increase is modest, and the incremental Nagelkerke *R* ^2^ of 0.0014 indicates a small gain in explained variance. This limited contribution is further reflected in model discrimination: the AUC improved only from 0.703 in Model 3 to 0.704 in Model 4, indicating that entropy added negligible discriminative power to the model despite its statistically significant independent association. Readers should interpret the statistical significance of entropy as evidence of a genuine, reproducible signal rather than as an indication of strong predictive or discriminative utility at the individual patient level. Because entropy was entered into a model already containing diagnosis count, which shares moderate correlation with entropy (*r* = 0.544), and the CCI, this modest, residual contribution is expected rather than anomalous. In methodological validation studies, the primary objective is to demonstrate that a new measure captures variance not accounted for by existing tools, rather than to maximise overall predictive performance. Under contemporary unitary frameworks of validity, construct validity is built incrementally from multiple strands of evidence rather than established by any single statistic [20, 21]. The consistent incremental contribution of entropy across all sensitivity analyses therefore contributes one such strand of discriminant evidence, discussed as follows.

A stratified analysis by diagnosis count provides further insight into the boundary conditions of this association. Entropy was not independently predictive among patients with fewer than seven diagnoses but showed a consistent, statistically significant association among patients with seven or more diagnoses. Because the stratum with the largest sample size (4–6 diagnoses, *n* = 8808) did not reach significance while smaller strata with higher diagnosis counts did, this pattern is consistent with the possibility that the incremental relevance of diagnostic dispersion becomes more apparent at higher levels of diagnostic burden, although formal interaction testing would be required to establish effect modification. A plausible explanation is that when a patient has only a small number of diagnoses, the practical difference in coordination demand between a concentrated and a dispersed diagnostic profile may be limited, regardless of entropy value. Conversely, this difference may become more meaningful as overall diagnostic volume increases, making diagnostic entropy most informative among patients with moderate‐to‐high diagnostic burden. This qualifies the motivating example presented in the Introduction: the coordination burden implied by diagnostic dispersion may be most relevant to patients with a substantial diagnostic burden, rather than universally across all levels of diagnostic complexity. An alternative, nonclinical explanation for this pattern is noted in the Limitations.

Why entropy predicts LOS independently of the CCI cannot be directly established with the present data, since the underlying mechanism, whether it reflects cross‐system coordination time costs or some other pathway, was not directly tested. One plausible explanation, consistent with the broader care fragmentation literature, is that prolonged hospitalisation in NCD general wards may be driven less by the severity of any single disease than by the time costs of coordinating care across multiple specialities [12, 13, 22, 23]. Under this hypothesis, high‐entropy patients would require input from multiple clinical teams, potentially face conflicting medication orders across systems and need results from multiple investigative streams before discharge planning can proceed, activities that mortality‐oriented severity indices are not designed to capture [10, 24]. This mechanistic account remains hypothesised rather than directly tested in the present study.

As noted above, diagnostic entropy differs from existing administrative‐data measures in the aspect of complexity it captures. Mortality‐weighted indices such as CCI and Elixhauser quantify severity but are insensitive to how diagnoses distribute across systems [7, 9, 10]. Simple diagnosis counts capture volume but cannot distinguish system‐concentrated from system‐dispersed diagnostic profiles with identical counts, despite their differing coordination demands [5, 11]. Diagnostic entropy complements these tools by quantifying cross‐system diagnostic dispersion directly and automatically from coded records, addressing a subdimension of comorbidity‐related complexity that neither volume nor weighted severity captures. Whether this dispersion corresponds to genuine care coordination burden, as opposed to a purely diagnostic phenomenon, is a question the present findings raise rather than answer. Prior work establishing that diagnostic diversity can be computed from ICD chapter distributions [16, 17] supports the feasibility of this approach; the present study extends it by testing predictive validity against a clinically relevant hospital outcome under rigorous statistical control. Similar associations between care fragmentation and hospital outcomes have been reported in US‐based national database studies [22, 23], suggesting that the empirical pattern observed here, whatever its underlying mechanism, is unlikely to be unique to the Chinese healthcare context.

The negative association between CCI and prolonged LOS in the fully adjusted model also warrants comment. This pattern was not confined to oncology patients. Notably, oncology patients had the highest median CCI and longest median LOS, but the weakest raw CCI–LOS correlation in the sample (Results). This finding is consistent with the possibility that hospitalisation duration in this subgroup may be shaped more by treatment‐cycle scheduling than by comorbidity‐weighted severity. However, confirming this explanation would require data on treatment intent and admission type, which were not available in the present dataset. This pattern does not indicate that comorbidity severity is clinically irrelevant. Rather, it is compatible with the conceptual distinction between CCI and diagnostic entropy, although it does not independently establish that distinction.

Beyond its predictive performance, diagnostic entropy offers several practical advantages as an administrative‐data measure. It requires no disease‐specific weighting, is computationally straightforward and is compatible with any version of ICD coding without recalibration. These properties make it readily applicable to routine discharge data across diverse settings [16, 17]. The use of ICD‐10 chapter‐level granularity, rather than three‐character codes, was a deliberate conceptual choice. Chapters provide a reproducible administrative approximation of broad diagnostic system domains and thus offer a natural, if imperfect, unit for measuring cross‐speciality coordination load. Finer granularity would introduce within‐speciality coding variation that is not relevant to the coordination construct. Full construct validation will require further strands of evidence beyond those presented here; convergent validity against alternative dispersion measures, criterion validity against nursing workload and known‐group validity across care settings are the logical next steps.

A critical next step for this line of research is to test whether diagnostic entropy corresponds to genuinely greater nursing complexity, rather than merely longer hospital stays. Nursing Minimum Datasets (NMDS), which capture standardised nursing diagnoses, nursing interventions and nursing‐sensitive outcomes generated during actual patient care, offer a promising avenue for this validation, with recent work showing that standardised nursing diagnoses can be linked to hospital outcomes and that nursing complexity independently predicts mortality, readmission and emergency department visits [25, 26]. Two further findings strengthen this direction: medical diagnosis count has been shown to predict nursing action count, which partially mediates LOS in paediatric patients [27], and frailty and nursing complexity interact to prolong hospital stay specifically in heart failure inpatients, the largest primary diagnosis category in our own sample [28]. Linking diagnostic entropy to NMDS‐derived measures of nursing complexity, alongside frailty and patient‐level vulnerability, would clarify whether cross‐system diagnostic dispersion reflects genuine nursing workload or overlaps with dimensions already captured through nursing documentation. Such validation, ideally prospective and multicentre, would determine whether entropy graduates from a hypothesis‐generating administrative signal to a validated indicator of nursing‐relevant care coordination complexity.

If diagnostic dispersion does reflect coordination burden, as hypothesised above, the findings would have implications for nursing management. Nurse staffing levels are robustly associated with patient outcomes, including mortality and LOS, across a large international evidence base [29–32]. Yet current staffing models in Chinese hospitals remain primarily based on bed numbers and nursing care levels, with limited consideration of cross‐system coordination complexity [33, 34]. Ward‐level mean diagnostic entropy could potentially serve as a supplementary complexity weight in staffing allocation decisions. Wards with consistently high mean entropy may benefit from a higher proportion of senior nurses with multisystem management competencies, given that high‐entropy patients are hypothesised to require coordination across multiple speciality teams. This proposal rests on diagnostic dispersion in administrative records rather than directly observed nursing time, and prospective studies linking ward entropy profiles to nursing workload measurements would need to precede any formal staffing recommendation.

A related consideration concerns the equity of coordination demands across patient subgroups. URRBMI patients constituted 83.6% of this sample, reflecting the predominantly rural and peri‐urban catchment area of the study hospital. The entropy distribution was similar across insurance types (URRBMI 83.7% vs. UEBMI 82.4% in the high‐entropy group), suggesting that cross‐system diagnostic dispersion is broadly distributed across the payer mix rather than concentrated in any one population. Household income and financial protection were not measured in this administrative dataset, but existing literature indicates that URRBMI enrollees typically have lower incomes and weaker financial protection than UEBMI enrollees, and continue to experience higher exposure to catastrophic health expenditure despite insurance integration [35–37]. If diagnostic dispersion is confirmed to reflect genuine coordination burden, tools for identifying coordination‐intensive patients may therefore be particularly relevant for protecting financially vulnerable subgroups, though this implication remains speculative given the absence of economic data in the present study.

### 4.1. Limitations

Several limitations should be noted in interpreting these findings. First, this was a single‐centre retrospective study, and the entropy distribution and its associations with LOS may not generalise to other hospitals, regions or health systems. Second, repeat admissions were present; although cluster‐robust estimation and first‐admission analyses produced similar findings, residual within‐patient dependence cannot be entirely excluded. Third, prolonged hospitalisation was used as an indirect outcome rather than a direct measure of care coordination or nursing workload. Fourth, administrative coding may vary in completeness and consistency across institutions and coders, and unmeasured factors such as functional status, social support, clinical severity and treatment characteristics may have influenced LOS. Fifth, the UEBMI subgroup was relatively small, and its less precise estimate should not be interpreted as evidence of an absent association. Sixth, entropy is mathematically constrained when diagnosis counts are low, limiting interpretation of the lower diagnosis‐count strata. Finally, the study was restricted to four broad NCD primary‐diagnosis categories, and the study period overlapped with ongoing diagnosis‐related group (DRG) payment reforms in China, which may have influenced coding practices and LOS management.

## 5. Conclusion

This study contributes discriminant evidence toward the construct validity of diagnostic entropy as an administrative‐data measure of cross‐system diagnostic dispersion in NCD inpatients. After controlling for both diagnostic volume and the CCI, entropy independently predicted prolonged hospitalisation, with consistent results across four sensitivity analyses. The findings suggest that cross‐system diagnostic dispersion represents a subdimension of comorbidity‐related complexity that existing comorbidity indices do not capture. If future research confirms the hypothesised link to care coordination burden, ward‐level diagnostic entropy, calculated from routine discharge data, could offer a practical and administratively feasible supplement to existing nursing workload tools, particularly in general wards managing patients with multisystem NCD. Full construct validation, as outlined above, will require prospective, multicentre research directly linking entropy to nursing workload and coordination indicators.

## Funding

This research was supported by the China Scholarship Council (Grant no. 202500560013).

Open access publishing facilitated by The University of Waikato, as part of the Wiley ‐ The University of Waikato agreement via the Council of Australasian University Librarians.

## Disclosure

The funders had no role in the study design, data collection, data analysis, data interpretation or manuscript preparation.

## Ethics Statement

This study was approved by the Institutional Review Board of The Third People’s Hospital of Yibin (Approval No: 2025042) and the Health Research Ethics Committee of the University of Waikato (Approval No: HREC(Health)2025#83). As the study utilised de‐identified retrospective administrative data, the requirement for individual informed consent was waived.

## Conflicts of Interest

The authors declare no conflicts of interest.

## Supporting Information

Additional supporting information can be found online in the Supporting Information section.

## Supporting information


**Supporting Information** Supporting Table S1 provides complete definitions, model roles and coding schemes for all variables used in the nested logistic regression models, including reference categories for categorical variables. Supporting Note S1 provides the full methodological rationale for the use of raw (non‐normalised) Shannon entropy rather than normalised entropy or the effective number of systems (2^*H*^) as the primary exposure measure in this study. STROBE checklist filled.

## Data Availability

The data that support the findings of this study are held by the hospital’s administrative department. Restrictions apply to the availability of these data, which were used under licence for the current study and are not publicly available due to patient confidentiality and institutional data governance policies. However, de‐identified data may be made available from the corresponding author upon reasonable request.
