# Supplementary material for: Diagnostic Entropy as an Administrative Measure of Cross‐System Diagnostic Dispersion in Noncommunicable Disease Inpatients: A Methodological Validation Study
Source: J Nurs Manag. 2026 Sep 20;2026:6726900. doi: 10.1155/jonm/6726900 (PMC13591114; doi:10.1155/jonm/6726900)
Supplement: Supplementary file 1 [file JONM-2026-6726900-s001.zip › STROBE checklist filled.docx]

**STROBE Statement — Checklist of items that should be included in reports of observational studies**

*Diagnostic Entropy as a Measure of Care Coordination Complexity in Non-Communicable Disease Inpatients: A Methodological Validation Study*

|  | **Item No.** | **Recommendation** | **Page No.** | **Relevant text from manuscript** |
| --- | --- | --- | --- | --- |
| **Title and abstract** | 1 | (a) Indicate the study's design with a commonly used term in the title or the abstract | 1 | *Title: "...A Methodological Validation Study". Abstract Methods: "A retrospective methodological validation study used inpatient discharge records from a tertiary hospital in southwest China, 2023–2025 (n=31,470)."* |
|  |  | (b) Provide in the abstract an informative and balanced summary of what was done and what was found | 1–2 | *Structured abstract with Background, Methods, Results, and Conclusions sections, summarising study aim, design, key findings (entropy OR=1.112, 95%CI 1.072–1.153, p<0.001), and conclusions.* |
| **Introduction** | | | | |
| **Background / rationale** | 2 | Explain the scientific background and rationale for the investigation being reported | 3–4 | *Introduction paragraphs 1–4: rising multimorbidity burden in NCD inpatients; CCI and Elixhauser indices summarise comorbidity burden but do not capture cross-system distribution; care fragmentation literature linking dispersed care with longer stays; Shannon entropy as a natural framework for distributional dispersion.* |
| **Objectives** | 3 | State specific objectives, including any prespecified hypotheses | 4 | *"We aim to provide preliminary evidence for the construct validity of diagnostic entropy by examining its incremental predictive value for prolonged hospitalisation beyond both diagnosis count and the CCI in a cohort of 31,470 NCD inpatients."* |
| **Methods** | | | | |
| **Study design** | 4 | Present key elements of study design early in the paper | 5 | *"This was a retrospective methodological validation study conducted at a tertiary general hospital in Sichuan Province, China."* |
| **Setting** | 5 | Describe the setting, locations, and relevant dates, including periods of recruitment, exposure, follow-up, and data collection | 5 | *Tertiary general hospital in Sichuan Province, China. Inpatient medical record data extracted from the hospital information system for three consecutive years (2023 to 2025).* |
| **Participants** | 6 | (a) Cross-sectional study—Give the eligibility criteria, and the sources and methods of selection of participants | 5 | *"All adult inpatients with a primary diagnosis falling within four major NCD categories were included: circulatory diseases (ICD-10 Chapter I), respiratory diseases (Chapter J), neoplasms (Chapter C), and endocrine diseases (Chapter E). Patients with missing primary diagnosis codes were excluded. The final sample comprised 31,470 records."* |
|  |  | (b) For matched studies, give matching criteria | — | *Not applicable (no matching).* |
| **Variables** | 7 | Clearly define all outcomes, exposures, predictors, potential confounders, and effect modifiers. Give diagnostic criteria, if applicable | 5–6 | *Methods sections "Diagnostic entropy calculation", "Charlson Comorbidity Index", and "Other variables" explicitly define: exposures (diagnostic entropy, diagnosis count, CCI); primary outcome (LOS>10 days); secondary outcome (log-transformed total cost); exploratory outcome (in-hospital mortality); covariates (age, sex, insurance, emergency admission, rural residence, primary diagnosis category, year).* |
| **Data sources / measurement** | 8* | For each variable of interest, give sources of data and details of methods of assessment (measurement). Describe comparability of assessment methods if there is more than one group | 5 | *All variables derived from hospital information system discharge records. "Diagnostic entropy was calculated using the Shannon entropy formula: H = −Σ pᵢ × log₂(pᵢ)..." "The CCI was calculated using the Quan et al. (2005) ICD-10 mapping, implemented via the comorbidity package (version 1.1.0) in R."* |
| **Bias** | 9 | Describe any efforts to address potential sources of bias | 5–6 | *ICD-10 Chapter Z and R codes excluded from entropy calculation as not representing definitive diagnoses; sensitivity analysis S2 including Z/R codes confirmed robustness. Multicollinearity addressed via VIF assessment with threshold <5. Three pre-specified sensitivity analyses (S1, S2, S3) addressing extreme values, coding rules, and insurance stratification.* |
| **Study size** | 10 | Explain how the study size was arrived at | 5 | *Complete enumeration of all eligible NCD inpatient discharges from the participating hospital over three consecutive years (2023–2025), yielding n=31,470. No formal sample size calculation was performed as this was a full-population administrative-data study.* |
| **Quantitative variables** | 11 | Explain how quantitative variables were handled in the analyses. If applicable, describe which groupings were chosen and why | 5–6 | *Entropy standardised to a z-score before entry into regression models; OR reflects change per one SD increase. Age also standardised. LOS dichotomised at 10 days (75th percentile of distribution, 22.3% of patients). Entropy additionally categorised into four groups (H=0; 0<H<1.0; 1.0≤H<1.5; H≥1.5) for descriptive analysis (Table 1).* |
| **Statistical methods** | 12 | (a) Describe all statistical methods, including those used to control for confounding | 6 | *Four nested binary logistic regression models. Model 1: demographics + clinical covariates. Model 2: + diagnosis count. Model 3: + CCI. Model 4: + standardised entropy. Spearman rank correlations among entropy, CCI, and diagnosis count. Model fit assessed via AUC and Nagelkerke R²; incremental R² from Model 3 to Model 4 quantifies entropy's additional contribution.* |
|  |  | (b) Describe any methods used to examine subgroups and interactions | 6 | *Sensitivity analysis S3 stratified the main regression model by insurance type (URRBMI vs UEBMI) to examine consistency across subgroups.* |
|  |  | (c) Explain how missing data were addressed | 5 | *Patients with missing primary diagnosis codes excluded at the eligibility stage. Secondary diagnosis fields without a valid ICD-10 code were treated as absent diagnoses and did not contribute to entropy calculation. No additional missingness in covariates.* |
|  |  | (d) Cross-sectional study—If applicable, describe analytical methods taking account of sampling strategy | — | *Not applicable (complete enumeration of eligible discharges; no sampling).* |
|  |  | (e) Describe any sensitivity analyses | 6 | *Three pre-specified sensitivity analyses: S1 excluded patients with LOS>30 days (n=148 excluded) to test influence of extreme values; S2 recalculated entropy including Z and R chapter codes to test robustness of coding exclusion; S3 stratified main analysis by insurance type to test consistency across subgroups.* |
| **Results** | | | | |
| **Participants** | 13* | (a) Report numbers of individuals at each stage of study | 7 | *"A total of 31,470 NCD inpatients were included across three years (2023: n=10,840; 2024: n=10,388; 2025: n=10,242)." Eligibility criteria yielded the analytic sample after excluding records with missing primary diagnosis codes.* |
|  |  | (b) Give reasons for non-participation at each stage | — | *Not applicable (administrative data; no participant-level non-participation).* |
|  |  | (c) Consider use of a flow diagram | — | *Not included; sample selection process is straightforward (all NCD inpatients with valid primary diagnosis codes over the 3-year study period) and fully described in the Participants section.* |
| **Descriptive data** | 14* | (a) Give characteristics of study participants and information on exposures and potential confounders | 7–8 | *Results paragraph "Sample description" reports demographic, clinical, and administrative characteristics. Table 1 provides full baseline characteristics stratified by diagnostic entropy group, including age, sex, insurance type, admission route, residence, LOS, total cost, mortality, CCI, diagnosis count, entropy, and year.* |
|  |  | (b) Indicate number of participants with missing data for each variable of interest | 5 | *All included records had complete data on the covariates used in regression (eligibility required valid primary diagnosis; secondary diagnosis fields without valid codes treated as absent diagnoses).* |
|  |  | (c) Cohort study—Summarise follow-up time | — | *Not applicable (cross-sectional administrative data; no follow-up).* |
| **Outcome data** | 15* | Cross-sectional study—Report numbers of outcome events or summary measures | 8 | *Prolonged hospitalisation (LOS>10 days) occurred in 7,022 patients (22.3% overall). High-entropy patients had higher rates of prolonged LOS (24.6% vs 14.5%). In-hospital mortality occurred in 174 patients (0.55%). Outcomes also tabulated by entropy group in Table 1.* |
| **Main results** | 16 | (a) Give unadjusted estimates and, if applicable, confounder-adjusted estimates and their precision. Make clear which confounders were adjusted for and why | 8–9 | *Table 3 reports ORs with 95% CIs for all variables across Models 1–4. Confounders explicitly listed with reference categories (Sex=Female; Insurance=UEBMI; Admission=Non-emergency; Residence=Urban; Primary diagnosis=Circulatory; Year=2023). Core result: entropy OR=1.112 (95%CI 1.072–1.153, p<0.001) in fully adjusted Model 4.* |
|  |  | (b) Report category boundaries when continuous variables were categorized | 6, 8 | *LOS dichotomised at >10 days (75th percentile). Entropy categories defined in Table 1 footnote: H=0; 0<H<1.0; 1.0≤H<1.5; H≥1.5.* |
|  |  | (c) If relevant, consider translating estimates of relative risk into absolute risk | 8 | *Absolute risks reported alongside ORs: high-entropy patients had LOS>10 days in 24.6% vs 14.5% in low-entropy patients (Table 1; Results, Entropy distribution).* |
| **Other analyses** | 17 | Report other analyses done—eg analyses of subgroups and interactions, and sensitivity analyses | 9 | *Three sensitivity analyses fully reported: S1 (excluding LOS>30 days): OR=1.103 (1.063–1.144, p<0.001); S2 (including Z/R codes): OR=1.122 (1.082–1.163, p<0.001); S3 (insurance-stratified): URRBMI OR=1.121 (p<0.001), UEBMI OR=1.052 (p=0.279).* |
| **Discussion** | | | | |
| **Key results** | 18 | Summarise key results with reference to study objectives | 10 | *Discussion paragraph 1: "After controlling for demographic characteristics, diagnosis count, and CCI simultaneously, diagnostic entropy remained independently associated with LOS>10 days (OR=1.112, 95%CI: 1.072–1.153, p<0.001). The association held across all three sensitivity analyses, with entropy ORs ranging from 1.103 to 1.122."* |
| **Limitations** | 19 | Discuss limitations of the study, taking into account sources of potential bias or imprecision. Discuss both direction and magnitude of any potential bias | 12 | *Six limitations explicitly enumerated: single-centre design; indirect outcome proxy (LOS as workload surrogate); residual confounding from unmeasured non-clinical patient factors; underpowered UEBMI subgroup (n=5,154); ICD coding quality variation; potential temporal confounding across the 3-year study period (year was controlled).* |
| **Interpretation** | 20 | Give a cautious overall interpretation of results considering objectives, limitations, multiplicity of analyses, results from similar studies, and other relevant evidence | 10–12 | *Discussion paragraphs 2–7: effect size carefully interpreted given the stringent analytic context (paragraph 2); mechanism related to coordination time costs (paragraph 3); diagnostic entropy positioned against CCI, Elixhauser, and diagnosis counts (paragraph 4); nursing staffing implications framed as preliminary (paragraph 5); equity implications calibrated against existing insurance literature (paragraph 6); methodological strengths and remaining validation needs outlined (paragraph 7).* |
| **Generalisability** | 21 | Discuss the generalisability (external validity) of the study results | 12 | *Limitation 1 explicitly addresses external validity: "this was a single-centre retrospective study; the generalisability of the entropy distribution and its predictive associations to other hospital types or regions requires further investigation." Multi-centre replication noted as a key next step in the Conclusion.* |
| **Other information** | | | | |
| **Funding** | 22 | Give the source of funding and the role of the funders for the present study and, if applicable, for the original study on which the present article is based | 13 | *"This research is supported by the China Scholarship Council (Grant No. 202500560013). The funders had no role in the study design, data collection, data analysis, data interpretation, or manuscript preparation."* |

**Give information separately for cases and controls in case-control studies and, if applicable, for exposed and unexposed groups in cohort and cross-sectional studies.*

*Note: An Explanation and Elaboration article discusses each checklist item and gives methodological background and published examples of transparent reporting. The STROBE checklist is best used in conjunction with this article (freely available on the Web sites of PLoS Medicine at http://www.plosmedicine.org/, Annals of Internal Medicine at http://www.annals.org/, and Epidemiology at http://www.epidem.com/). Information on the STROBE Initiative is available at www.strobe-statement.org.*
