# Supplementary material for: Diagnostic Entropy as an Administrative Measure of Cross‐System Diagnostic Dispersion in Noncommunicable Disease Inpatients: A Methodological Validation Study
Source: J Nurs Manag. 2026 Sep 20;2026:6726900. doi: 10.1155/jonm/6726900 (PMC13591114; doi:10.1155/jonm/6726900)
Supplement: Supplementary file 1 [file JONM-2026-6726900-s001.zip › Supplementary_Note_S1_Rationale for using raw (non-normalized) Shannon entropy.docx]

**Supplementary Note S1. Rationale for using raw (non-normalized) Shannon entropy.**

Diagnostic entropy in this study was calculated as raw Shannon entropy, H = −Σ pᵢ × log₂(pᵢ), rather than normalized entropy (H divided by the maximum possible entropy given the total number of ICD-10 chapters, i.e., log₂(22) for the full ICD-10 chapter structure) or the effective number of systems (2^H).

This choice was made for the following reason. Normalizing entropy by the full 22-chapter ICD-10 structure would implicitly assume that all 22 chapters are equally likely to co-occur within a single patient's diagnostic profile. This assumption does not reflect clinical reality in an NCD inpatient population: some chapter combinations are far more clinically plausible and common than others. For example, comorbid circulatory (Chapter I) and endocrine (Chapter E) diagnoses, such as hypertension and type 2 diabetes, are frequent in this population, whereas combinations such as circulatory and dermatological (Chapter L) diagnoses are rare. Normalizing against a theoretical maximum that assumes uniform co-occurrence across all 22 chapters would therefore compress the realistic range of entropy values observed in this population into an artificially narrow portion of the 0–1 normalized scale, reducing interpretability and discriminative resolution.

Raw entropy avoids this assumption. It remains directly interpretable in bits, reflects the actual observed distribution of diagnoses across chapters for each patient, and does not require an arbitrary or population-specific choice of a theoretical maximum.

We acknowledge that normalized entropy or the effective number of systems may offer complementary interpretability in future work, particularly for studies comparing entropy across different patient populations or diagnostic contexts where the realistic range of chapter co-occurrence may differ substantially from the present NCD inpatient sample. Researchers applying diagnostic entropy in other clinical populations should consider whether normalization is appropriate given the co-occurrence structure of that population.
