# Supplementary material for: Diagnostic Entropy as an Administrative Measure of Cross‐System Diagnostic Dispersion in Noncommunicable Disease Inpatients: A Methodological Validation Study
Source: J Nurs Manag. 2026 Sep 20;2026:6726900. doi: 10.1155/jonm/6726900 (PMC13591114; doi:10.1155/jonm/6726900)
Supplement: Supplementary file 1 [file JONM-2026-6726900-s001.zip › Supplementary_Table_S1_variable_definitions.docx]

**Supplementary Table S1.** Definitions, roles, and coding of variables used in the nested logistic regression models.

| **Variable** | **Role in model** | **Type** | **Coding / unit** |
| --- | --- | --- | --- |
| LOS>10 days | Primary outcome | Binary | 1 = length of stay > 10 days; 0 = length of stay ≤10 days |
| Diagnostic entropy (standardised) | Exposure of interest (Model 4) | Continuous, standardised | Shannon entropy H = −Σ pᵢ × log₂(pᵢ), in bits; z-standardised (mean=0, SD=1) before entry into regression models |
| Charlson Comorbidity Index (CCI) | Covariate (Models 3–4) | Continuous | Weighted score using the Quan (2005) ICD-10 mapping (comorbidity R package, v1.1.0) |
| Diagnosis count | Covariate (Models 2–4) | Continuous (count) | Number of valid ICD-10 codes per patient; observed range 1–16 |
| Age (standardised) | Covariate (Models 1–4) | Continuous, standardised | Years; z-standardised (mean=0, SD=1) |
| Sex | Covariate (Models 1–4) | Binary | 1 = Male; 0 = Female (reference) |
| Insurance type | Covariate (Models 1–4) | Binary | 1 = URRBMI; 0 = UEBMI (reference) |
| Admission route | Covariate (Models 1–4) | Binary | 1 = Emergency; 0 = Non-emergency (reference) |
| Residence | Covariate (Models 1–4) | Binary | 1 = Rural; 0 = Urban (reference) |
| Primary diagnosis category | Covariate (Models 1–4) | Categorical (3 dummy variables) | Reference category: Circulatory disease (ICD-10 Chapter I). Dummy variables: Respiratory disease (Chapter J), Neoplasms (Chapter C), Endocrine disease (Chapter E) |
| Year | Covariate (Models 1–4) | Categorical (2 dummy variables) | Reference category: 2023. Dummy variables: 2024, 2025 |

*Note. Model 1 included demographic and clinical covariates only (age, sex, insurance type, admission route, residence, primary diagnosis category, year). Model 2 added diagnosis count. Model 3 further added the Charlson Comorbidity Index (CCI). Model 4 added standardised diagnostic entropy. All continuous predictors entered as standardised (z-score) variables are indicated as such; unstandardised continuous variables (diagnosis count, CCI) are entered in their natural units. LOS = length of stay; URRBMI = Urban and Rural Resident Basic Medical Insurance; UEBMI = Urban Employee Basic Medical Insurance.*
